# Hedonic eating in Prader–Willi syndrome is associated with blunted PYY secretion

**DOI:** 10.1080/16546628.2017.1297553

**Published:** 2017-05-02

**Authors:** A. E. Rigamonti, S. Bini, F. Piscitelli, A. Lauritano, V. Di Marzo, C. Vanetti, F. Agosti, A. De Col, E. Lucchetti, G. Grugni, A. Sartorio

**Affiliations:** ^a^ Department of Clinical Sciences and Community Health, University of Milan, Milan, Italy; ^b^ Endocannabinoid Research Group, Institute of Biomolecular Chemistry, Consiglio Nazionale delle Ricerche, Pozzuoli, Italy; ^c^ Department of Medical Biotechnology and Translational Medicine, University of Milan, Milan, Italy; ^d^ Experimental Laboratory for Auxo-Endocrinological Research, Istituto Auxologico Italiano, IRCCS, Milan and Piancavallo (VB), Italy; ^e^ Division of Auxology, Istituto Auxologico Italiano, IRCCS, Piancavallo (VB), Italy

**Keywords:** Palatable food, PYY, ghrelin, CCK, endocannabinoids, Prader-Willi syndrome, hunger, satiety

## Abstract

Hedonic and homeostatic hunger represent two different forms of eating: just for pleasure or following energy deprivation, respectively. Consumption of food for pleasure was reported to be associated with increased circulating levels of both the orexigenic peptide ghrelin and some specific endocannabinoids in normal-weight subjects and patients with morbid obesity. To date, the effects of palatable food on these mediators in Prader–Willi syndrome (PWS) are still unknown. To explore the role of some gastrointestinal orexigenic and anorexigenic peptides and endocannabinoids (and some related congeners) in chocolate consumption, we measured changes in circulating levels of ghrelin, cholecystokinin (CCK), peptide YY (PYY), anandamide (AEA), 2-arachidonoyl-glycerol (2-AG), palmitoylethanolamide (PEA) and oleoylethanolamide (OEA) in eight satiated adult PWS patients after consumption of chocolate and, on a separate day, of a non-palatable isocaloric food with the same macronutrient composition. Evaluation of hunger and satiety was also performed by visual analogic scale. The anticipatory phase and the consumption of food for pleasure were associated with decreased circulating levels of PYY. An increase in PEA levels was also observed. By contrast, circulating levels of ghrelin, CCK, AEA, 2-AG and OEA did not differ before and after the exposure/ingestion of either chocolate or non-palatable foods. Hunger and satiety were similar in the hedonic and non-palatable sessions. In conclusion, when motivation to eat is promoted by highly palatable foods, a depressed post-prandial PYY secretion is observed in PWS. Although preliminary, these findings seem to hypothesize a possible role of PYY agonists in the management of PWS patients.

**Abbreviations:** AEA, Anandamide; 2-AG, 2-arachidonoyl-glycerol; CB_1_, cannabinoid receptor type 1; OEA, oleoylethanolamide; PEA, palmitoylethanolamide; PWS: Prader-Willi syndrome; VAS, visual analog scales

## Introduction

In animals and humans, eating is stimulated not only by the need to restore energy homeostasis (homeostatic hunger), but also by the rewarding properties of highly palatable foods, mainly fatty and/or sugar-sweet meals, despite a state of satiety and positive energy balance (hedonic hunger) [1]. Scientific interest is focused on investigating the physiological and pathophysiological mechanisms underlying hedonic hunger in order to characterize new pharmacological targets to counteract obesity and other eating disorders.

Animal studies have demonstrated that homeostatic and hedonic eating are regulated by different, although overlapping, neural pathways (at central level) and hormonal responses (at peripheral level), which involve several appetite-regulating substances [2,3]. A huge number of gastrointestinal endocrine cells produces and secretes satiety hormones in response to food consumption and digestion. These hormones, including cholecystokinin (CCK), peptide YY (PYY) and glucagon-like peptide 1 (GLP-1), suppress homeostatic hunger and promote satiety [4]. In addition to these anorexigenic peptides, there is an orexigenic peptide produced by the stomach, named ghrelin, which is capable to stimulate not only homeostatic, but also hedonic hunger [5,6]. These effects are mediated by activation of the receptor, GHS-R1a, which is expressed not only in the hypothalamus (controlling homeostatic hunger), but also in tegmental and mesolimbic areas (involved in food-related reward) [5].

Food intake is also stimulated by the endocannabinoids anandamide (AEA) and 2-arachidonoyl-glycerol (2-AG), two lipid mediators that act mainly at central level by activating the cannabinoid type 1 (CB_1_) receptors, which in turn are widely distributed in several brain areas, including those involved in the homeostatic and hedonic control of feeding [7,8].

Hedonic hunger has been also investigated in humans. In this context, Monteleone et al. [9–11] showed that normal-weight, healthy subjects, when administered with palatable food, exhibited increased circulating levels of both ghrelin and 2-AG, and that this post-prandial response was disrupted in women with anorexia nervosa, an eating disorder associated with altered secretion of many appetite-regulating substances. In another work, circulating levels of CCK were reported to be decreased when normal-weight healthy subjects were exposed to palatable food [12]. Furthermore, Rigamonti et al. [13] demonstrated that patients with essential morbid obesity exhibited increased circulating levels of ghrelin, AEA and 2-AG after anticipation and consumption of chocolate, with no differences in PYY and GLP-1 concentrations. Again in obese individuals, Monteleone et al. [14] reported that plasma levels of 2-AG increased after eating a favourite food, and decreased after eating the non-favourite food; the levels of AEA, instead, decreased progressively in non-hedonic eating, whereas they showed a decrease only immediately after the exposure to the favourite food, followed by a subsequent increase towards pre-meal levels.

Prader–Willi syndrome (PWS) is a neurogenetic disorder, characterized, in addition to other clinical signs and symptoms, by hyperphagia resulting in severe obesity in adolescence and adulthood if no strict dietetic control is adopted in association with modifications in life style [15,16]. Several clinical studies have been carried out in PWS patients to understand the pathophysiological mechanisms underlying the hyperphagic attitude and, particularly, to identify the presumptive causative role of any orexigenic or anorexigenic gut peptide.

As demonstrated by several studies, PWS children and adults exhibit markedly elevated fasting and post-prandial levels of ghrelin in plasma, thus suggesting that the hypersecretion of this peptide could be implicated in hyperphagia and delayed meal termination in PWS [17–21]. However, this view seems to be contradicted by the failure of ghrelin suppression to reduce food intake in PWS [22–25]. On the contrary, only a few studies have investigated post-prandial GLP-1, CCK and PYY in PWS, besides reporting conflicting results [21,26–30]. Interestingly, an unexpected increase of the post-prandial responses of PYY and GLP-1 to fast feeding was observed in adult PWS patients, suggesting a potential involvement of these anorexigenic peptides in the pathophysiology of this eating disorder [31].

To the best of our knowledge, post-prandial concentrations of orexigenic and anorexigenic peptides such as ghrelin, CCK and PYY have not been assessed in PWS patients in the context of hedonic vs homeostatic hunger. Additionally, to date, no one has measured post-prandial levels of endocannabinoids in PWS patients.

Thus, in order to investigate the potential association of endocannabinoids and gastrointestinal peptides, specifically, ghrelin, PYY and CCK, with hedonic eating in PWS, we have measured here changes in the circulating levels of these appetite-regulating substances before and after the exposure to and consumption of chocolate, a well-known highly palatable food, in satiated PWS patients.

## Materials and methods

### Subjects

Eight male patients with PWS, aged 19–42 yr (mean ± SD = 35.6 ± 8.3 yr), having a mean BMI ± SD 38.8 ± 10.1 kg/m^2^, hospitalized for a short period of time for periodical re-evaluation of their clinical conditions, were enrolled into the study. The subjects were recruited from the Division of Auxology at Istituto Auxologico Italiano, IRCCS, Verbania, Italy.

All patients showed the typical PWS clinical phenotype. Cytogenetic analysis revealed interstitial deletion of the proximal long arm of the paternally derived chromosome 15 in all patients. All PWS subjects had previously undergone GH treatment, withdrawn in all cases at least 2 years before starting the study protocol. A mild mental retardation was present in all individuals and, in this respect, the requirement for participating in the study was a score over the cut-off value of 24 in the Mini Mental State Examination (MMSE) [32].

Each participant enrolled in the study was requested to fulfil the following conditions:

1. to positively respond to the following question:‘Is chocolate one of your most favorite foods that you would eat also when satiated, just for pleasure?’;
2. to give a palatability score ≥8 for chocolate, being the administered scale ranging from 0 (not palatable) to 10 (maximally palatable).

Exclusion criteria included previous diagnosis of any disease affecting the endocrine system and metabolism (apart from PWS), chronic use of medications affecting metabolism and/or appetite, ≥5.0 kg weight change during the 3 months preceding study participation, and allergies to or stated dislike of the components of the test meal (see below), None of the subjects was a marijuana smoker, an alcohol consumer or heavy cigarette smoker, conditions known to affect circulating levels of endocannabinoids.

All participants were fully informed of the nature and procedures of the study. Therefore, each subject was aware that, in the first session of the experimental protocol, he would have eaten chocolate.

### Study design

The experiment used a within-subject repeated-measure design in which each volunteer served as his own control, identical to that used by Rigamonti et al. [12]. All subjects were tested two times with an interval in-between the tests of at least 7 days. A single-blind latin-square crossover design could not be applied because of the experimental needs of evoking the anticipatory effect of palatable food and of administering a non-palatable food with the same macronutrients and calories of the consumed palatable food (see below).

On the first test session, participants arrived at our Clinical Investigation Unit at 08.30 h after a 12-h fast. At 09.00 h, they were asked to rate their hunger and satiety on visual analogue scales (VAS) that used a 10-cm line with labels at the extremities indicating the most negative and the most positive ratings; immediately afterwards, an iv catheter was inserted into an antecubital vein to collect a first blood sample (time (T) = 0); the catheter was connected to a saline solution, which was slowly infused to keep it patent through the entire experimental session. Then, the subjects received a breakfast of 200 kcal, with 77% carbohydrates, 10% proteins and 13% fats. Immediately after breakfast (consumed within 10 min), they rated again their hunger and satiety by means of VAS. Further blood samples were drawn (T10 and T30 at 10 min and 30 min, respectively). After 1 h from the start of the study, the subjects were told that they would receive chocolate. Immediately afterwards, each participant was exposed to the palatable food for 10 min. During this time, he could smell and see the food but could not eat it. At the end of the exposure, each participant was asked to rate his hunger, satiety by means of VAS. Blood samples were drawn at 60 min and 70 min (i.e. T60 and T70). Then, the subject was free to eat the palatable food (see below for details) within 10 min. Additional blood samples were drawn immediately after the exposure to the palatable food (T80) and at 100 min (T100), 130 min (T130), 160 min (T160) and 190 min (T190); at the same time points, they rated again their hunger and satiety by means of VAS.

At the end of the session, the amount of food eaten by each participant was calculated by weighing the residual food and subtracting it from the initial amount of food provided, and then the calories eaten were calculated.

On the second test session, carried out at least 7 days later, participants underwent the same experimental procedures of the first experimental session except for the fact that they were exposed to non-palatable food and had to eat an amount of it with the same macronutrient composition and an equal quantity of calories as the palatable food they ate in the previous session within 10 min.

During the food exposure (specifically, from T60 to T70), a total of 20 pictures of chocolate-made foods and of landscapes and nature were shown in the session with chocolate and non-palatable food, respectively.

Palatable and non-palatable foods

The palatable food was a milk-chocolate tablet (200 g for a total of 1000 kcal with 61.4% carbohydrates, 7.9% proteins, and 30.7% fat), served in a dish from which the subject was free to eat until he became satiated (for a maximum corresponding to the whole chocolate tablet).

The non-palatable food, which was identified by all participants as non-desirable just for pleasure (specifically, with a palatability score <2) consisted of bread and butter, which were combined ad hoc to provide the same macronutrients and calories of the consumed chocolate. Calorie and nutrient contents of palatable and non-palatable foods were calculated by using the information reported on the labels of each packaged food (chocolate tablet and butter). To calculate calorie and nutrient content of bread, we obtained the recipe from the baker who made it.

To maintain a stable daily caloric intake of the in-hospital PWS patients, the amount of foods administered at lunch and dinner of the experimental days was proportionally reduced to account for the calories of the test meals (i.e. chocolate or non-palatable food).

### Evaluation of body composition

Anthropometric characteristics were evaluated during the screening period. BMI was calculated from measured height and weight. Fat-free mass (FFM) and fat mass (FM) were evaluated by bioelectrical impedance analysis (Human-IM Scan, DS-Medigroup, Milan, Italy).

### Blood sampling and biochemical measurements

Blood was collected in tubes with or without anticoagulant (EDTA). Plasma or serum was separated by centrifugation and stored at −20 C.

Total plasma ghrelin level, including both octanoylated and des-octanoylated ghrelin, was measured by a commercially available RIA (Millipore, Saint Charles, MO, USA). The sensitivity of the method was 93 pg/ml; intra- and interassay coefficients of variation (CVs) were 10.0 and 14.7% respectively. The concentration range was 10,496–6718 pg/ml and the recovery percentage 90%.

Total plasma PYY level, including both PYY_1-36_ and PYY_3–36_, was measured by a commercially available RIA (Millipore, Saint Charles, MO, USA). The sensitivity of the method was 10 pg/ml; intra- and interassay CVs were 2.9 and 7.1% respectively. The concentration range was 1117–1430 pg/ml and the recovery percentage 96%.

Serum CCK, precisely CCK_6–33_ solphate (100% cross-reactivity), was measured by a commercially available radioimmunoassay kit (EuroDiagnostica, Malmö, Sweden) after an extraction procedure, as indicated in the instructions provided by the manufacturer. The sensitivity of the method was 0.3 pmol/l; intra- and interassay CVs were 5.5 and 13.7%, respectively. The concentration range was 0,78–25 pmol/l and the recovery percentage 80%.

Serum insulin concentration was determined by chemiluminescent immunometric assay using a commercial kit (Immulite 2000, DPC, Los Angeles, CA, USA). The sensitivity of the method was 2 µIU/ml; intra- and interassay CVs were 22–38% and 14–23%, respectively.

Serum glucose level was measured by the glucose oxidase enzymatic method (Roche Diagnostics, Monza, Italy).

Plasma levels of AEA, 2-AG, oleoylethanolamide (OEA) and palmitoylethanolamide (PEA) were determined by isotopic dilution-liquid chromatography-mass spectrometry, as described previously [13].

### Statistical analysis

As conventionally established, the sample size was determined for giving 80% power at the .05 level of significance (two-sided). The expected mean difference of circulating levels of ghrelin 20 min after exposure to palatable vs non-palatable food (200 pg/ml) and the estimated standard deviation of the same variable (150 pg/ml)) were deducted by the results reported in the work by Rigamonti et al. [13].

The Sigma Stat 3.5 statistical software package was used for data analysis. GraphPad Prisma 5.0 software was used for plotting data.

The Shapiro–Wilk test showed that all parameters were normally distributed.

Results are reported as mean ± SD (standard deviation). The responses in glucose, insulin, ghrelin, PYY, CCK, AEA, 2-AG, PEA, OEA and VAS scores for hunger and satiety were evaluated as absolute values for each experimental session of eating (breakfast + chocolate and breakfast + non-palatable).

All parameters (ghrelin, PYY, CCK, VAS scores for hunger and satiety, glucose and insulin) were compared within each experimental session of eating (breakfast + chocolate and breakfast + non-palatable-food) over sampling times (intra-group analysis) and between the two experimental sessions of eating for any sampling time (inter-group analysis) by using a two-way ANOVA with repeated measures (with the two factors time and session and the interaction time × session), followed by the *post hoc* Bonferroni’s test, which was used to compare responses after breakfast (i.e. T10, T30 and T60 vs 0 min) and the responses after chocolate or non-palatable food (i.e. T70, T80, T100, T130, T160 and T190 vs 60 min) for both experimental sessions of eating (i.e. breakfast + chocolate and breakfast + non-palatable-food). The same statistical test was applied for analysing the responses in endocannabinoids and related congeners (AEA, 2-AG, PEA and OEA) only after the second part of the experimental session (i.e. T70, T100, T130 and T190 vs 60 min for both experimental sessions of eating). A two-way ANOVA, followed by the *post hoc* Bonferroni’s test, was used to compare the ratios of circulating ghrelin levels to those of PYY (ghrelin/PYY) at T0 and T80 in both sessions of eating. A level of significance of p < 0.05 was used for all data analyses.

## Results

### Body composition and other clinical information

The Table 1 reports data of body composition in our population of PWS patients. Importantly, body weight did not significantly change in each PWS subject, when the first (breakfast + chocolate) and second (breakfast + non-palatable-food) sessions of eating were compared (data not shown).

**Table 1.** Demographic and clinical characteristics of the PWS subjects enrolled in the study.

	PWS Subjects
Number (n.)	8
Age (yrs)	35.6 ± 8.3
BMI (kg/m^2^)	38.8 ± 10.1
FFM (kg)	55.3 ± 11.4
FFM (%)	58.7 ± 3.8
FM./kg)	39.5 ± 12.1
FM (%)	41.3 ± 3.8

PWS = Prader–Willi syndrome; BMI = body mass index; FM = free fat mass; FM = fat mass.

### Calorie ingestion

As expected, the mean values of calories and macronutrients of palatable and non-palatable foods were not significantly different (data not shown). PWS subjects ate 181.3 ± 25.9 g of chocolate (range: 150–200 g), which correspond to 925.0 ± 103.5 kcal (range: 800–1000 kcal).

### Circulating levels of gastrointestinal peptides: ghrelin, PYY and CCK

Table 2 reports the statistical results of the time × session repeated measures ANOVA applied to each parameter (ghrelin, PYY and CCK).

**Table 2.** Statistical results of the time × session repeated measures ANOVA applied to each variable investigated.

	Factor		
Variable	Time	Session	Time × Session
Ghrelin	F(9/54)=1.32	F(1/6)=0.97	F(9/54)=1.47
CCK	F(4/24)=1.98	F(1/6)=0.56	F(4/24)=1.92
PYY	F(9/54)=6.32*	F(1/6)=3.82*	F(9/54)=1.08*
2-AG	F(4/24)=2.03	F(1/6)=1.24	F(4/24)=1.58
AEA	F(4/24)=3.22*	F(1/6)=1.08	F(4/24)=1.68
PEA	F(4/24)=0.96	F(1/6)=2.43*	F(4/24)=2.11
OEA	F(4/24)=3.17	F(1/6)=1.09	F(4/24)=1.14
Hunger	F(9/54)=3.98*	F(1)=2.15	F(9/54)=2.78
Satiety	F(9/54)=3.04*	F(1/6)=2.01	F(9/54)=2.88
Glucose	F(9/54)=2.69*	F(1/6)=1.15	F(9/54)=2.02
Insulin	F(9/54)=3.06*	F(1/6)=0.98	F(9/54)=2.14

*: p<0.05.

#### Ghrelin

**Figure 1.**
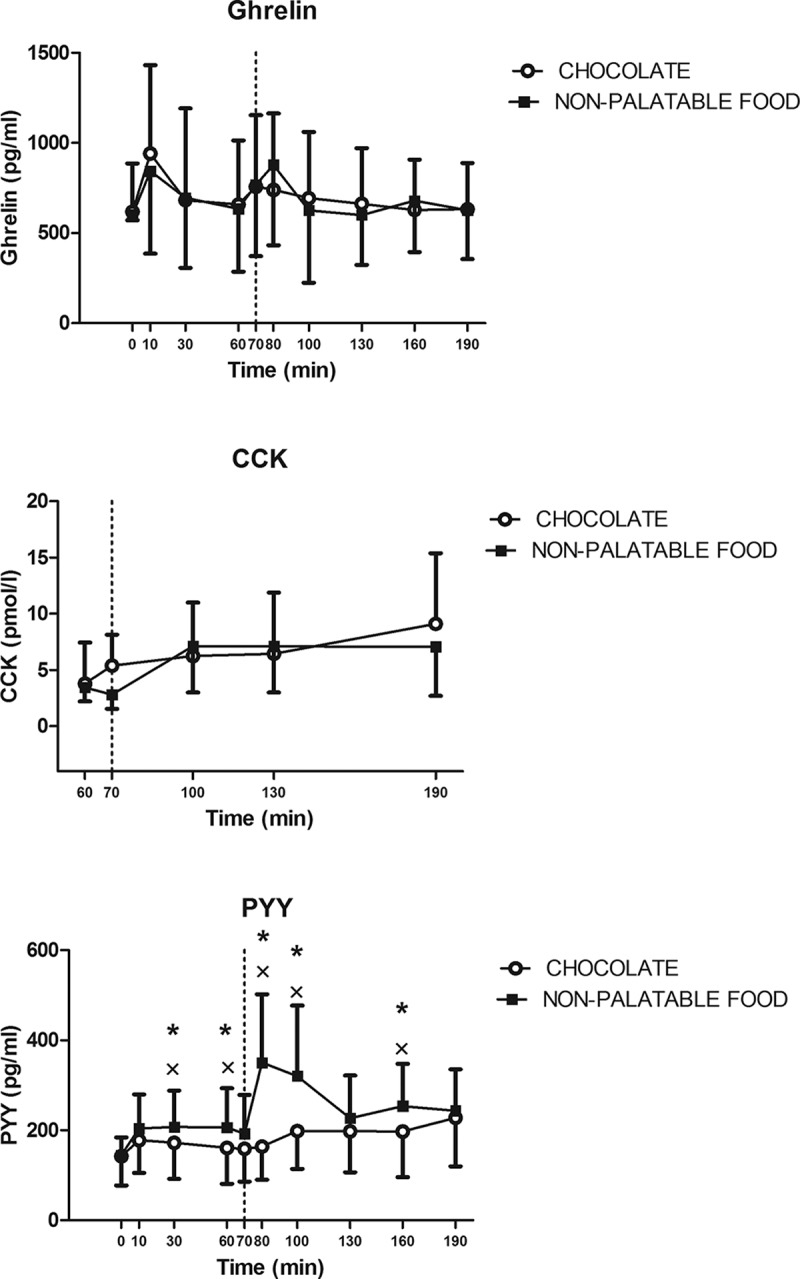
Changes of circulating levels of ghrelin (top panel), CCK (middle panel) and PYY (bottom panel) in PWS patients after breakfast (at the left of the dotted vertical line, i.e. T0-T70) and chocolate or non-palatable meal (at the right of the dotted vertical line, i.e. T70-T190) during the hedonic and non-palatable sessions of eating, respectively. Breakfast was consumed from T0 to T10, while chocolate or non-palatable meal from T70 to T80 after a sensorial exposure of the foods and view of pictures of chocolate-made foods (in the hedonic session) or landscapes and nature (in the non-palatable session) from T60 to T70. See the text for further details. Values are expressed as mean ± SD. * p < 0.05 vs the corresponding time point of the non-palatable session; × p < 0.05 vs the corresponding T0 or T60 value.

Circulating levels of ghrelin did not change significantly over the sampling times and between the two experimental sessions of eating (Figure 1).

#### PYY

There was a statistically significant increase in circulating levels of PYY at 30 and 60 min (vs 0 min after the breakfast, p < 0.01) and at 80, 100 and 160 min after non-palatable food in PWS patients administered with the breakfast + non-palatable session, whereas no such difference was found in those administered with the breakfast + chocolate session (intra-group analysis). Furthermore, the PWS subjects, tested with the hedonic session, when compared with the non-palatable session, exhibited significantly lower plasma concentrations of PYY at 30, 60, 80, 100 and 160,min (p < 0.01) (inter-group analysis) (Figure 1).

#### CCK

Circulating levels of CCK did not change significantly over sampling times and between the two experimental sessions of eating (Figure 1).

#### Ratio ghrelin/PYY

**Figure 2.**
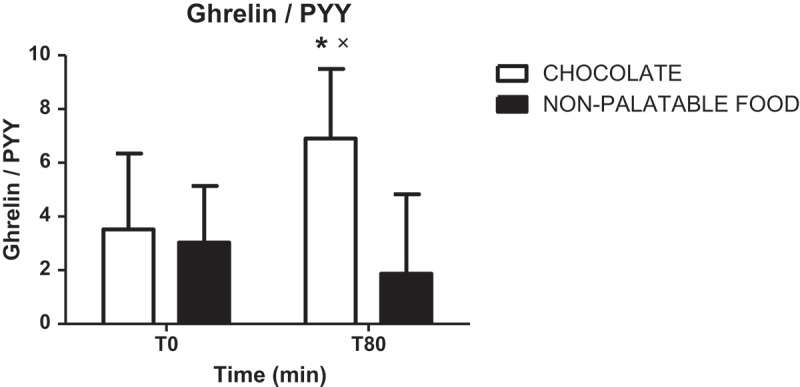
Ratios of circulating ghrelin levels to those of PYY (ghrelin/PYY) at T0 and T80 in PWS patients administered with breakfast + chocolate or breakfast + non-palatable-food. Values are expressed as mean ± SD. * p < 0.05 vs T80 of the non-palatable session; × p < 0.05 vs T0 of the hedonic session.

There was no statistically significant difference in the ratio ghrelin/PYY at T0 among PWS patients administered with breakfast + chocolate or breakfast + non-palatable-food. On the contrary, the ratio ghrelin/PYY at T80 was significantly higher than that at T0 during hedonic eating (p < 0.05) and at the same time point after non-palatable food (p < 0.05). Although not statistically significant, the ratio ghrelin/PYY at T80 after non-palatable food was lower than that at T0 during the same session of eating (Figure 2).

### Circulating levels of endocannabinoids and related mediators: AEA, 2-AG, PEA and OEA

Table 2 reports the statistical results of the time × session repeated measures ANOVA applied to each parameter (AEA, 2-AG, PEA and OEA).

#### AEA

**Figure 3.**
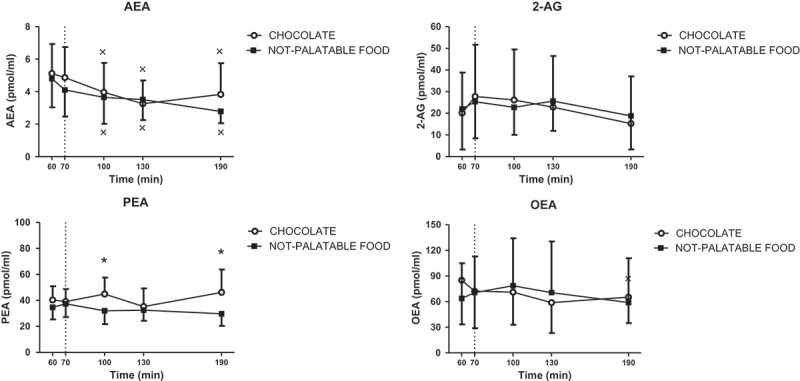
Changes of circulating levels of anandamide (AEA, top left panel), 2-arachidonoyl-glycerol (2-AG, top right panel), palmitoylethanolamide (PEA, bottom left panel) and oleoylethanolamide (OAE, bottom right panel) in satiated PWS patients before (i.e. T60-T70) and after (i.e. T60-T190) chocolate or non-palatable meal during the hedonic and non-palatable sessions of eating, respectively. Chocolate or non-palatable meal was consumed from T70 to T80 after a sensorial exposure of the foods and view of pictures of chocolate-made foods (in the hedonic session) or landscapes and nature (in the non-palatable session) from T60 to T70. See the text for further details. Values are expressed as mean ± SD. *p < 0.05 vs the corresponding time point of the non-palatable session; × p < 0.05 vs the corresponding T60 value.

There was a statistically significant decrease in circulating levels of AEA at 100, 130 and190 min (vs 60 min, p < 0.05) in PWS patients administered with the breakfast + chocolate session and also with the breakfast + non-palatable-food session (intra-group analysis), with no difference between the two sessions (inter-group analysis); (Figure 3).

#### 2-AG

Circulating levels of 2-AG did not change significantly over sampling times and between the experimental sessions of eating (Figure 3).

#### PEA

Circulating levels of PEA were significantly higher at 100 and 190 min in PWS patients administered with the breakfast + chocolate session than the breakfast + non-palatable session (p < 0.05) inter-group analysis), with no significant time-related changes (intra-group analysis); (Figure 3).

#### OEA

Circulating levels of OEA did not change significantly over sampling times and between the experimental sessions of eating (Figure 3).

### VAS scores: hunger and satiety

Table 2 reports the statistical results of the time × session repeated measures ANOVA applied to each parameter (hunger and satiety).

#### Hunger

**Figure 4.**
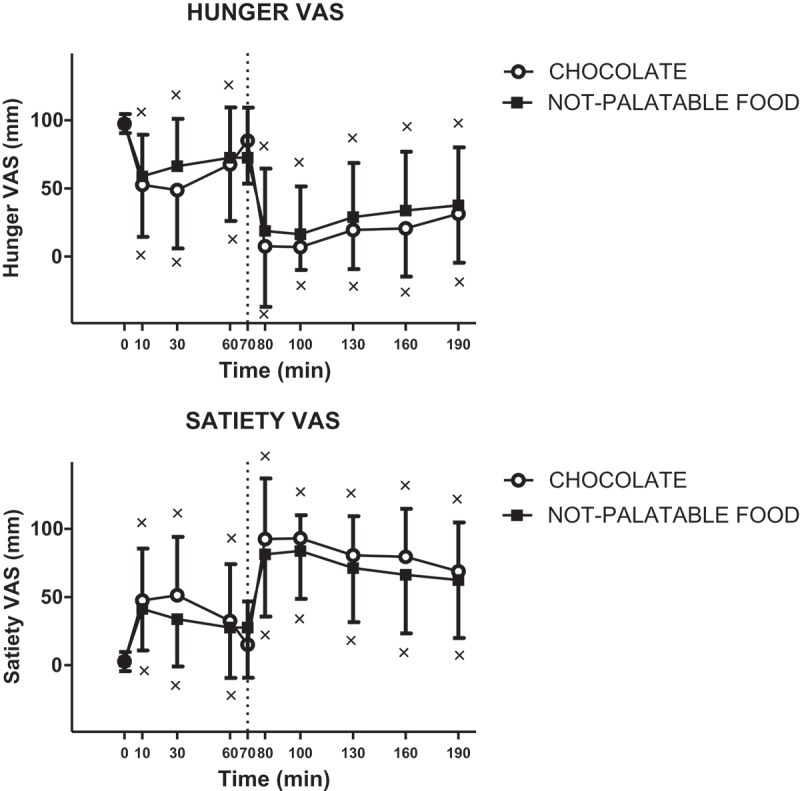
Changes of VAS ratings of hunger (top panel) and satiety (bottom panel) in PWS patients after breakfast (at the left of the dotted vertical line, i.e. T0-T70) and chocolate or non-palatable meal (at the right of the dotted vertical line, i.e. T70-T190) during the hedonic and non-palatable sessions of eating, respectively. Breakfast was consumed from T0 to T10, while chocolate or non-palatable meal from T70 to T80 after a sensorial exposure of the foods and view of pictures of chocolate-made foods (in the hedonic session) or landscapes and nature (in the non-palatable session) from T60 to T70. See the text for further details. Values are expressed as mean ± SD. × p < 0.05 vs the corresponding T0 or T60 value.

There was a statistically significant decrease in hunger VAS scores at 10, 30 and 60 min (vs 0 min after the breakfast, p < 0.01) for both experimental sessions and at 80, 100, 130, 160 and 190 min (vs 60 min after the chocolate or non-palatable food in PWS patients administered with the breakfast + chocolate and the breakfast + non-palatable-food sessions, respectively (intra-group analysis), with no difference between the two sessions (inter-group analysis)) (Figure 4).

#### Satiety

There was a statistically significant increase in satiety VAS scores at 10, 30 and 60 min (vs 0 min after the breakfast, p < 0.01) for the breakfast + chocolate and breakfast + non-palatable-food sessions and at 80, 100, 130, 160 and 190 min (vs 60 min after the chocolate or non-palatable food) for both sessions (intra-group analysis), with no difference between the two sessions (inter-group analysis) (Figure 4).

### Metabolic parameters: glucose and insulin

Table 2 reports the statistical results of the time × session repeated measures ANOVA applied to each parameter (glucose and insulin).

#### Glucose

**Figure 5.**
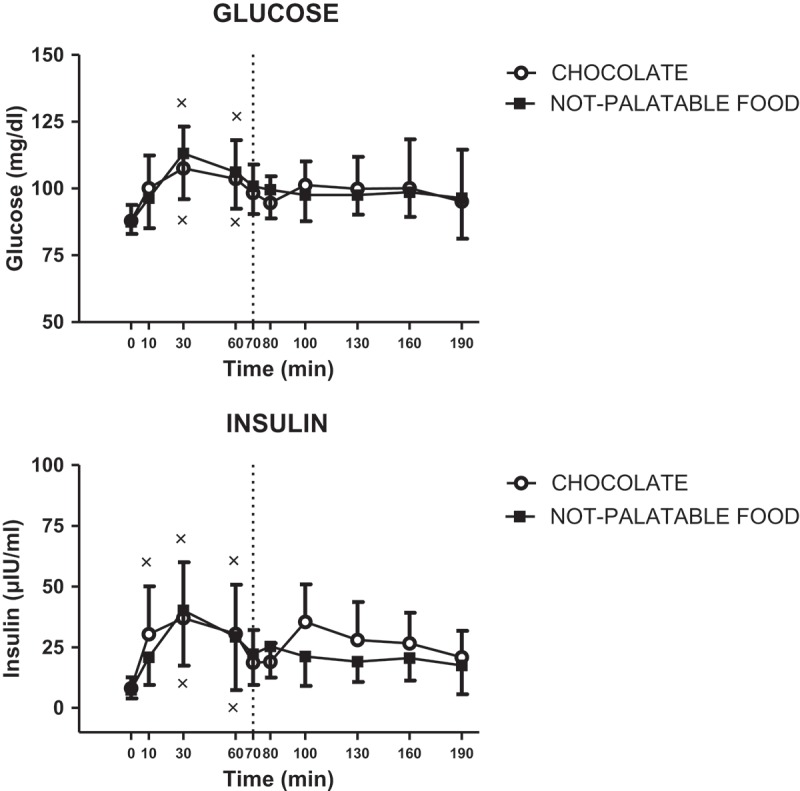
Changes of circulating levels of glucose (top panel) and insulin (bottom panel) in PWS patients after breakfast (at the left of the dotted vertical line, i.e. T0-T70) and chocolate or non-palatable meal (at the right of the dotted vertical line, i.e. T70-T190) during the hedonic and non-palatable sessions of eating, respectively. Breakfast was consumed from T0 to T10, while chocolate or non-palatable meal from T70 to T80 after a sensorial exposure of the foods and view of pictures of chocolate-made foods (in the hedonic session) or landscapes and nature (in the non-palatable session) from T60 to T70. See the text for further details. Values are expressed as mean ± SD. × p < 0.05 vs the corresponding T0 or T60 value.

Administration of both experimental sessions of eating (i.e. breakfast + chocolate or breakfast + non-palatable-food) evoked an identical statistically significant increase in glucose concentrations at 30 and 60 min (vs 0 min after breakfast, p < 0.01) (intra-group analysis), with no difference between the two sessions (inter-group analysis) (Figure 5).

#### Insulin

Administration of both experimental sessions of eating (i.e. breakfast + chocolate or breakfast + non-palatable-food) evoked an identical statistically significant increase in insulin concentrations at 10, 30 and 60 min (vs 0 min after breakfast, p < 0.01) (intra-group analysis), with no difference between the two sessions (inter-group analysis) (Figure 5).

## Discussion

The main finding of the present study carried out in PWS patients was that circulating levels of PYY were persistently found depressed during the entire hedonic session (breakfast + palatable-food session), which included the administration of a satiating breakfast and, one hour after, a chocolate tablet, which was initially served for stimulating an intense sensorial experience and then freely eaten. By contrast, increased PYY secretion was observed during the breakfast + non-palatable-food session, in which the chocolate tablet was replaced by an isoenergetic non-palatable meal with the same macronutrient composition (i.e. bread and butter). It is noteworthy that during the breakfast+ non-palatable-food session, two statistically significant peaks in circulating PYY levels occurred after breakfast (at T30-T60) and non-palatable food (at T80-T100), being the second response in PYY higher than the first one presumably because of the more caloric content in the non-palatable food than in the breakfast. The blunted PYY secretion in our PWS subjects, observed in the hedonic session of eating, was (perhaps) already present at T0 before starting the experiment.

In our PWS subjects, the persistent inhibition of PYY secretion during the entire hedonic session likely reflects the orexigenic state occurring in the cephalic phase (or, alternatively, the anticipatory phase) before consumption of highly palatable food, when all (both lean and obese) individuals think about, see and/or smell the food but do not eat it yet [33]. The anticipatory effect of highly palatable food is, obviously, a crucial aspect of our experimental protocol: specifically, the participants in the present study knew that they would have eaten chocolate in the first experimental session, because of our need to balance the caloric content and macronutrient composition of the non-palatable food to that of the palatable one (see also ‘Materials and methods’). However, at the beginning of the hedonic session of eating and after the exposure to the palatable food, such blunted PYY secretion seems to be a distinctive ‘paradoxical’ response in PWS, which is not present in lean subjects and patients with essential morbid obesity [9,13]). In fact, different from these individuals, who underwent a similar protocol, based on the findings obtained in the present study one might hypothesise that PWS subjects, who were aware that they would have eaten chocolate in that day, and, hence, were likely to experience an intense sensorial exposure to this palatable food (T60-T70), showed an inhibited PYY secretion possibly to facilitate the ensuing ingestion of pleasurable food (i.e. chocolate) and to block an early meal termination.

Paradoxically, this secretory pattern of PYY (i.e. the blunted PYY secretion) might be the only mechanisms through which PWS patients can eat for pleasure. In fact, PWS individuals have been demonstrated in many clinical studies to exhibit higher (fasting and post-prandial) ghrelin levels than lean subjects or patients with essential morbid obesity [17–21], levels that cannot increase further following exposure to palatable food.

This argumentation is supported by the present study showing no significant changes in ghrelin secretion throughout the entire protocol, a not surprising finding since circulating levels of ghrelin were reported to be not suppressed by food intake in PWS adults [17,20].

Rather than considering either PYY or ghrelin as single biochemical parameter, the ratio ghrelin/PYY seems to be of more relevant endocrinological and also pharmacological interest. In fact, in the present study, the ratio ghrelin/PYY at T80 after chocolate, which corresponds to the *climax* of the hedonic experience, was higher than that at the same time point after non-palatable food, confirming the occurrence of an orexigenic state in PWS patients administered with a palatable food (see also above). The ratio ghrelin/PYY (high for decreased PYY levels and/or increased ghrelin levels) might be a correlate of activation of the hedonic pathway of food intake. Although it might be proposed as a marker of development of hyperphagia in PWS, with important clinical and therapeutic implications, further studies are mandatory to confirm these intriguing hypotheses.

A limitation of the present study, however, concerns the lack of evaluation of des-octanoylated ghrelin (UAG). In fact, UAG is known to induce a negative energy balance by decreasing food intake and delaying gastric emptying *via* the hypothalamus [34]. Concerning our findings, further investigation isneeded, in order to better understand the role of the different forms of circulating ghrelin in hedonic eating in PWS.

Unexpectedly, in the present study, only a slow progressive increase in CCK secretion (not significant) was found after the second part of the two sessions of eating, without any difference between consumption of palatable and non-palatable meals. Few clinical studies have investigated the effects of a meal on CCK secretion in PWS, reporting conflicting results when compared to a control group [29,35–37]. Nevertheless, Monteleone et al. [12] have demonstrated that, in satiated normal-weight subjects, there is no post-prandial increase in circulating levels of CCK after administration of palatable food, suggesting that eating for the rewarding properties of a highly pleasurable food is also promoted by an absent or insufficient CCK-mediated ‘anorexigenic brake’. To date, we are not aware of the exact reason of the unchanged circulating levels of CCK in our PWS patients tested with the non-palatable food, such as reduced caloric content or inappropriate macronutrient composition of the administered (palatable or non-palatable) meals, few sampling times or methodological differences in CCK assay. Therefore, any speculation regarding the role of CCK in hedonic eating in PWS should await further clinical studies on this issue, taking also into account the availability of CCK agonists in pre-clinical pharmacological research [38].

In the present study, hunger and satiety VAS scores were congruent with ingestion of meals (breakfast or palatable/non-palatable food). We would have expected lower and higher values in hunger and satiety VAS score, respectively, after hedonic than non-palatable session, being PYY secretion depressed. However, this was not the case. A possible reason of this discrepancy may be the subjective measurement of these parameters (which are probably more strongly influenced also by the syndrome itself), as well as the wide variability and scarce reproducibility associated with any experimental method to measure appetite [39].

Different from the results obtained in lean subjects [9] and patients with essential morbid obesity [13,14], the findings of the present study do not point to an important role of the endocannabinoids AEA and 2-AG, and of the non-endocannabinoid AEA congener, OEA, in the modulation of hedonic eating in PWS. Specifically, in our study circulating levels of AEA, 2-AG and OEA were not significantly different between the two sessions with the exposure to chocolate or non-palatable food. By contrast, circulating levels of PEA were significantly higher at T100 and T190 in PWS patients administered with chocolate than non-palatable food. Finally, there was a significant decrease of circulating levels of AEA at T100, T130 and T190 in both sessions (i.e. breakfast + chocolate and breakfast + non-palatable-food), without any time-related differences for the other compounds 2-AG, PEA and OEA. These negative data regarding the endocannabinoids AEA and 2-AG are, however, in agreement with the lack of changes in ghrelin levels in hedonic eating in PWS patients, as discussed above, thus corroborating previous evidence on the existence of direct correlations between ghrelin secretion and endocannabinoid production [9,13].  They might also indicate the lack of any cross-talk between endocannabinoids and PYY, whose levels in our previous study in essential obese volunteers [13] did not change.

To date, there is scarce evidence that the peroxisome proliferator-activated receptor α (PPAR-α) and the two PPAR-α ligands OEA and PEA are implicated in the rewarding effects associated with food intake [40]. Therefore, it is difficult to interpret the statistically significant increase of circulating levels of PEA in PWS after exposure to chocolate, when compared to those in breakfast + non-palatable-food session. We can only speculate that, particularly at T190, when this increase was stronger, this response might represent an adaptive mechanism to counteract, via activation of PPAR-α, the peripheral inflammatory and metabolic (i.e. steatosis) consequences of exaggerated hedonic eating. These results are different from those reported in the study by Monteleone et al. [9,14] in normal-weight and obese subjects, respectively, in whom circulating levels of OEA and PEA were found to be similar following consumption of either favourite or non-favourite food, whereas in the study by Rigamonti et al. [13], carried out in patients with essential morbid obesity, circulating levels of OEA, but not PEA, were significantly higher at T60 before exposure to chocolate. Further studies in animals and humans are mandatory to understand the physiological role and the pathophysiological implications of endogenous PPAR-α ligands, including PEA and OEA, in food intake and, generally, reward.

Finally, in the present study the decline of circulating levels of AEA after ingestion of palatable or non-palatable food, already described by Rigamonti et al. [13] in patients with essential morbid obesity, might be due to circadian changes in the levels of this compound, previously demonstrated in the rat brain [41]. Alternatively, since PWS patients were still insulin-responsive, this finding is more likely due to post-prandial insulin-induced inhibition of plasma AEA levels, already observed by Di Marzo et al. [42].

## Conclusions

Clearly different from normal-weight healthy subjects [9] and patients with essential morbid obesity [13,14], when motivation to eat is generated by the availability of highly palatable food and not just by food deprivation, a depressed post-prandial PYY secretion, at least theoretically able to re-activate central rewarding system, was found in PWS patients. In this context, the role of total ghrelin and endocannabinoids seems to be negligible. Taking into account these preliminary observations, while patients suffering from essential morbid obesity might actually be treated with ghrelin receptor and CB_1_ neutral antagonists in order to block hyperphagia induced by palatable foods, the use of PYY agonists appears to be instead one of the most appropriate pharmacological strategy in PWS, allowing to reinforce the insufficient post-prandial anorexigenic response when PWS patients are exposed to palatable foods [31]. Although the present report should be considered preliminary, it might serve as a pilot study to be validated in future studies with larger cohorts.

## Availability of data and materials

All data from this study are included in the figures and the table of the manuscript.
